# New murine Niemann-Pick type C models bearing a pseudoexon-generating mutation recapitulate the main neurobehavioural and molecular features of the disease

**DOI:** 10.1038/srep41931

**Published:** 2017-02-07

**Authors:** Marta Gómez-Grau, Júlia Albaigès, Josefina Casas, Carme Auladell, Mara Dierssen, Lluïsa Vilageliu, Daniel Grinberg

**Affiliations:** 1 Department of Genetics, Faculty of Biology, University of Barcelona, 08028 Barcelona, Spain; 2 Institut de Biomedicina de la UB (IBUB)-Institut de Recerca Sant Joan de Déu (IRSJD), 08028 Barcelona, Spain; 3 Centre for Biomedical Research on Rare Diseases (CIBERER), 08028 Barcelona, Spain; 4 Cellular & Systems Neurobiology, Systems Biology Programme, Centre for Genomic Regulation (CRG), Barcelona Institute of Science and Technology, 08003 Barcelona, Spain; 5 Research Unit on BioActive Molecules (RUBAM), Departament de Química Biomèdica, Institut de Química Avançada de Catalunya (IQAC-CSIC), 08034 Barcelona, Spain; 6 Department of Cell Biology, Faculty of Biology, University of Barcelona, 08028 Barcelona, Spain; 7 Institute of Neurosciences, University of Barcelona, 08028 Barcelona, Spain; 8 Pompeu Fabra University (UPF), 08003 Barcelona, Spain

## Abstract

Niemann-Pick disease type C (NPC) is a rare neurovisceral disease caused mainly by mutations in the *NPC1* gene. This autosomal recessive lysosomal disorder is characterised by the defective lysosomal secretion of cholesterol and sphingolipids. No effective therapy exists for the disease. We previously described a deep intronic point mutation (c.1554-1009 G > A) in *NPC1* that generated a pseudoexon, which could be corrected at the cellular level using antisense oligonucleotides. Here, we describe the generation of two mouse models bearing this mutation, one in homozygosity and the other in compound heterozygosity with the c.1920delG mutation. Both the homozygotes for the c.1554-1009 G > A mutation and the compound heterozygotes recapitulated the hallmarks of NPC. Lipid analysis revealed accumulation of cholesterol in the liver and sphingolipids in the brain, with both types of transgenic mice displaying tremor and ataxia at 7–8 weeks of age. Behavioural tests showed motor impairment, hyperactivity, reduced anxiety-like behaviour and impaired learning and memory performances, features consistent with those reported previously in NPC animal models and human patients. These mutant mice, the first NPC models bearing a pseudoexon-generating mutation, could be suitable for assessing the efficacy of specific splicing-targeted therapeutic strategies against NPC.

Niemann-Pick disease type C (NPC; OMIM 257220) is a rare autosomal recessive, lysosomal and multisystemic neurodegenerative disorder caused by mutations in either the *NPC1* or *NPC2* gene. Mutations in NPC1, a protein located at the late endosomal/lysosomal limiting membrane, account for approximately 95% of NPC cases, while mutations in NPC2, a soluble cholesterol-binding protein found in the lysosomal lumen, are responsible for the remaining 5%. The loss of function of these proteins elicits an accumulation of unesterified cholesterol and sphingolipids in the late endosomes/lysosomes. Although the full function of the NPC1 and NPC2 proteins is still unclear^1^,^2^, it is thought that NPC1 and NPC2 work together to promote cholesterol export from late endosomes/lysosomes^1^. NPC is also described as a neurovisceral disorder, as it affects visceral organs such as the liver, spleen and lungs in addition to the central and peripheral nervous systems. Clinical presentation of the disease is extremely heterogeneous, with an age of onset ranging from the perinatal period until well into adulthood and the majority of patients dying between 10 and 25 years of age^3^. In about 90% of patients, progressive neurodegeneration is the dominant feature, with cerebellar ataxia, dysarthria, dysphagia and progressive dementia also typically observed. Cataplexy, seizures and dystonia are other quite common features.

The *NPC1* gene (OMIM 607623) is located on the long arm of chromosome 18 (18q11-q12) and is composed of about 56 kilobases. It consists of 25 exons encoding a 1,278-amino acid glycoprotein that is composed of 13 transmembrane domains, a sterol-sensing domain and a cytoplasmic C-terminal lysosomal targeting motif^4^,^5^. More than 380 mutations have been reported to date for this gene (HGMD Professional, 2015.4), including missense and nonsense mutations, as well as splicing, deletions and insertions. No effective treatment exists for the disease, although some positive effects have been described for the drug miglustat^6^ (OGT 918, *N*-butyl-deoxynojirimycin) and for cyclodextrin^7^ treatments, either in patients or animal models.

A metabolic and neurological phenotype mimicking human NPC disease has been described in Balb/C mice^8^, which was the first mouse model of NPC and has been used for most of the NPC studies in mice. New NPC models have been generated more recently, such as the conditional *Npc1*^*flox*^ KO mice^9^ and the chemically-induced *Npc1*^*nmf164*^ mice^10^. Mouse models with specific point mutations have also been created^11^,^12^.

In this study, we generated two homozygous NPC mouse models bearing specific mutations: the *Npc1*^*imagine*^ mouse carrying the deep intronic human mutation c.1554-1009 G > A, which we had previously observed in a Spanish NPC patient^13^, and the *Npc1*^*pioneer*^ mouse bearing the human mutation c.1920delG. We also generated compound heterozygous mice carrying both these mutations. The corresponding heterozygous mice from which we bred all the mice for this study were created by Ozgene and kindly provided to us by the Addi and Cassi Fund (http://addiandcassi.com/). We applied behavioural methods to determine the onset and development of the major symptoms of NPC in the mouse models. Molecular, biochemical and histological tests were also performed to assess splicing patterns and lysosomal storage. Our results showed that these novel NPC mouse models mimicked the main pathological features of human NPC, rendering them useful for testing new and approved therapeutic approaches. Moreover, the *Npc*^*imagine*^ mouse, which, to the best of our knowledge, is the first NPC model bearing a pseudoexon-generating mutation, could be suitable for assessing the efficacy of specific splicing-targeted therapeutic strategies against NPC.

## Results

### Generation of homozygous and compound heterozygous mice

The murine *Npc1* gene contains 25 exons and shares a high homology at the DNA (83.4% identity and similarity) and protein (86.4% identity and 93.5% similarity) levels with the human *NPC1* gene. To generate a mouse model mimicking the phenotype of NPC patients with the mutations c.1554-1009 G > A and c.1920delG, Ozgene generated two strains of mice, each bearing one of these two *NPC1* mutations in heterozygosity, and named them “Imagine” (I, c.1554-1009 G > A) and “Pioneer” (P, c.1920delG). To generate the c.1554-1009 G > A (I) mutation in mice, the whole mouse *NPC1* intron 9 was replaced with the human *NPC1* intron 9 containing the mutation. The change of a G base to an A in humans generates an alternative donor splice site, which in turn produces aberrant splicing that gives rise to a 194-base pair (bp) pseudoexon and a frameshift. The c.1920delG (P) mutation in exon 12 yields a truncated protein due to a frameshift. This mutation is situated at the beginning of the SSD domain, which is important for cholesterol binding. Heterozygous male and female mice were bred to obtain the homozygous animals *Npc1*^imagine/imagine^ (I/I) and *Npc1*^pioneer/pioneer^ (P/P), and the compound heterozygous mice, *Npc1*^imagine/pioneer^ (I/P).

### Birth rate and lifespan of mutant *Npc1* mice

The *Npc1*^pioneer/pioneer^ mice presented a very low birth rate. Only a few homozygous mutant animals (2%) were born from all the crosses (+/+, 72 mice; +/P, 98 and P/P, 5) and most of them were females, precluding any further characterisation of these animals. The *Npc1*^*imagine/imagine*^ mice presented a birth rate of 18%, and the outcome of the crosses was: +/+: 65; +/I: 125; I/I: 43. For the compound heterozygotes, crosses between two heterozygous mice yielded the following: +/+, 40 mice; +/I, 43; +/P, 45; and I/P, 36, i.e., a birth rate of 22% for I/P mice. The I/I and I/P mice had a shorter lifespan than WT animals, with an average of 9 weeks.

### Splicing pattern analysis in *Npc1* ^*imagine*^ homozygous mice

**Figure 1:**
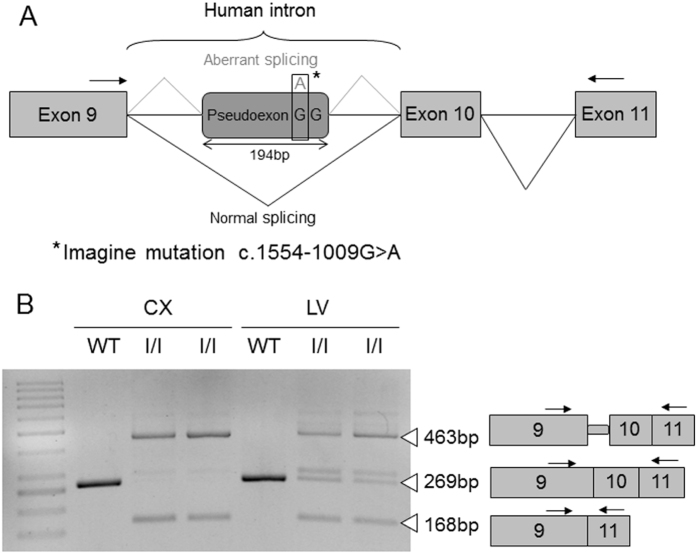
Splicing pattern generated by the c.1554-1009 G > A (Imagine) mutation. (**A**) Scheme of part of the *Npc1* Imagine allele, between exons 9 and 11, containing the human intron 9 with the mutation and the pseudoexon generated by the DNA change. (**B**) WT and alternative transcripts detected by RT-PCR (using the primers indicated by arrows) in the brain cortex (CX) and liver (LV) of WT and I/I mice.

To determine if the replacement of a complete murine intron 9 with the homologous human one bearing the c.1554-1009 G > A (I) mutation caused the aberrant splicing pattern observed in human patients carrying the mutation, RNA was extracted from the liver and brain cortex of *Npc1*^*imagine/imagine*^ mice and a specific RT-PCR experiment was performed using primers that hybridised with exons 9 and 11 (Fig. 1A).

As seen in Fig. 1B, a multiple-band pattern was observed in both tissues, which corresponded to different alternatively spliced transcripts. In the brain, WT mice only had the transcript corresponding to the normally spliced product, whereas two alternative transcripts were observed in the homozygous *Npc1*^*imagine*^ mice. The larger fragment (463 bp) included the 194-bp pseudoexon, while the smaller one (168 bp) corresponded to a transcript in which exon 10 was skipped. Similar results were obtained in the liver. However, two additional minor bands were observed in the homozygous *Npc1*^*imagine*^ animals, one of which corresponded to the WT transcript.

### Analysis of the NPC1 protein

**Figure 2:**
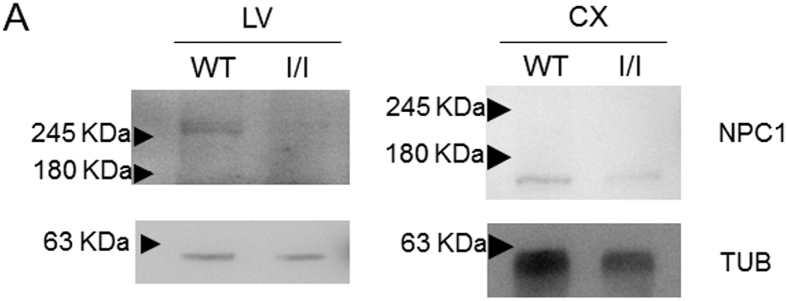
Western blot analysis of NPC1 protein expression in the (**A**) liver and (**B**) brain cortex isolated from WT and *Npc1*^*imagine/imagine*^ mice. Tubulin was used as the loading control.

To check for the presence of the NPC1 protein in the liver and brain cortex of *Npc1*^*imagine/imagine*^ mice, we performed western blot analysis with an anti-NPC1 antibody (Fig. 2). No band was observed in the liver of homozygous *Npc1*^*imagine*^ mice, but a faint band of apparently the same molecular weight as that of the WT protein was observed in the brain cortex.

### Sphingolipid and cholesterol analysis in mouse tissues

To determine whether the *Npc1*^*imagine*^ homozygous and compound heterozygous mice had a similar lipid storage phenotype as that observed in NPC patients and other NPC mouse models, we analysed sphingolipid levels in the brain and cholesterol amounts in the liver of WT, I/I and I/P mice.

**Figure 3:**
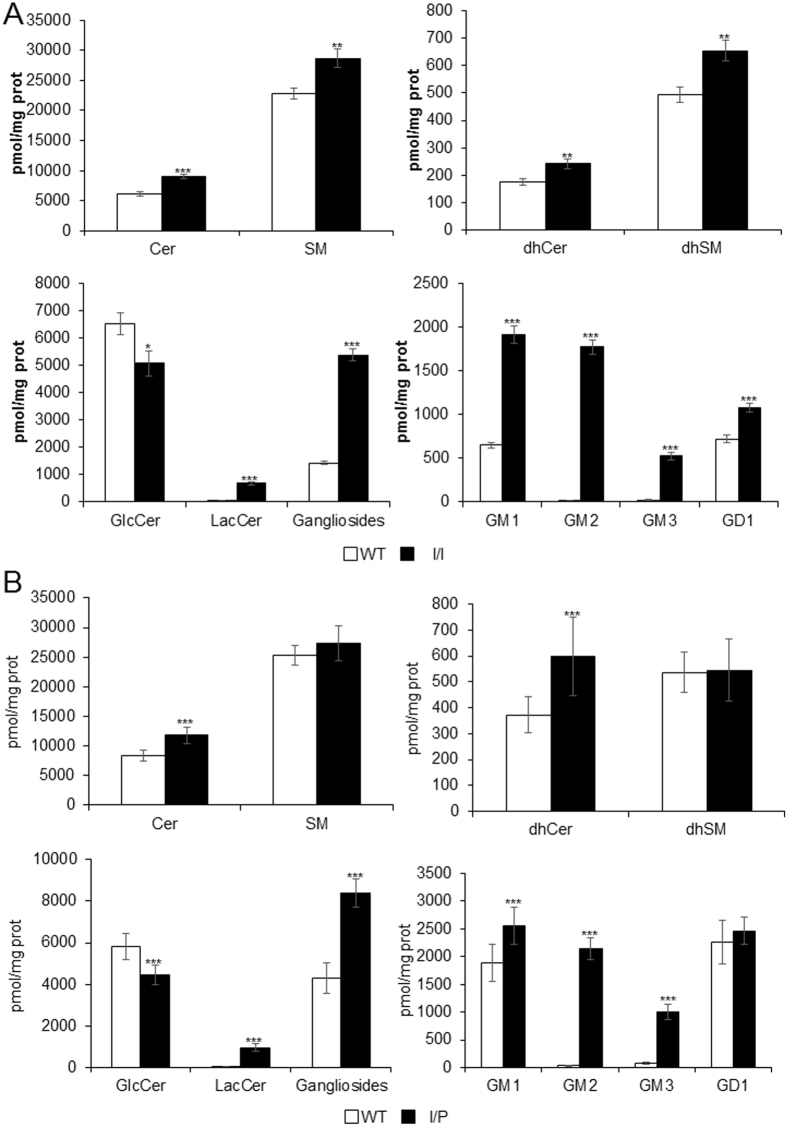
Brain lipid storage in (**A**, black) I/I and (**B**, black) I/P mice compared to (WT, white) wild-type mice. Data are expressed as mean ± SD. *p < 0.05, **p < 0.01 and ***p < 0.001. Cer, ceramide; SM, sphingomyelin; dhCer, dihydroceramide; dhSM, dihydrosphingomyelin; GlcCer, glucosylceramide; LacCer, lactosylceramide.

The I/I (Fig. 3A) and I/P (Fig. 3B) animals showed a significant accumulation of dihydroceramide (dhCer), lactosylceramide (LacCer) and gangliosides, particularly GM2 and GM3, and reduced monohexosylceramide (MHC, glucosyl and galactosylceramide) levels compared to WT. Additionally, a slight increase in ceramide (Cer) levels was also observed.

**Figure 4:**
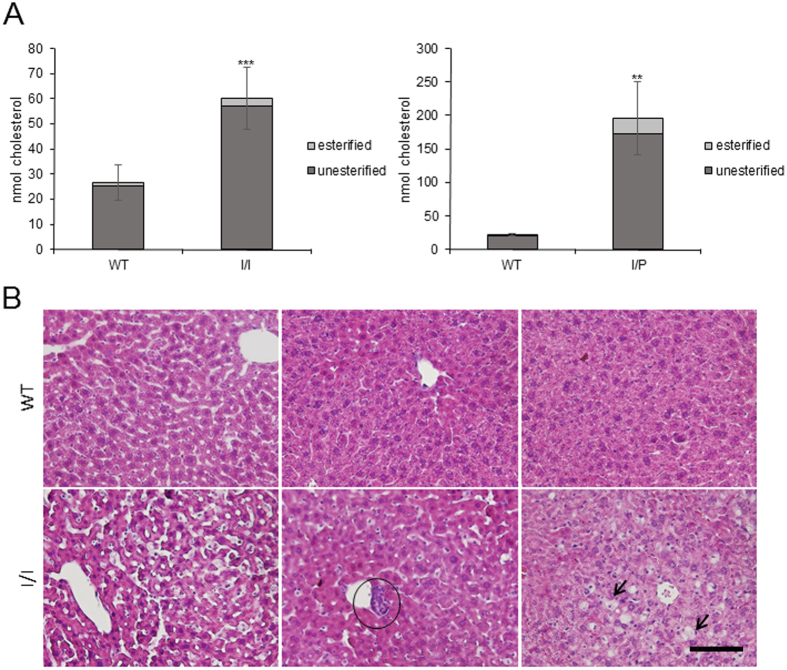
(**A**) Total hepatic cholesterol. Stacked bar chart showing quantification of esterified and unesterified cholesterol in WT and I/I or I/P mice. Data are shown as mean ± SD. **p < 0.01. (**B**) Histological analysis of liver tissue. Foamy macrophages in hepatocytes of I/I mice are indicated by black arrows. Neutrophil infiltration is marked with a black circle. Scale bar, 100 μm.

A significant accumulation of free and total cholesterol in the liver of both I/I and I/P mice was observed by gas chromatography-mass spectrometry (Fig. 4A). A similar result was obtained with the Amplex Red Cholesterol assays (not shown).

Histological analyses of liver tissue were performed to detect lipid levels in *Npc1*^*imagine/imagine*^ and WT mice. The mutant mice showed accumulation of lipids in vacuole-like inclusions (Fig. 4B, indicated by black arrows), as well as macrophage infiltration (Fig. 4B, indicated by a black circle), a different organisation of hepatocytes and sinusoid swelling.

### Cerebellar neuropathology

**Figure 5:**
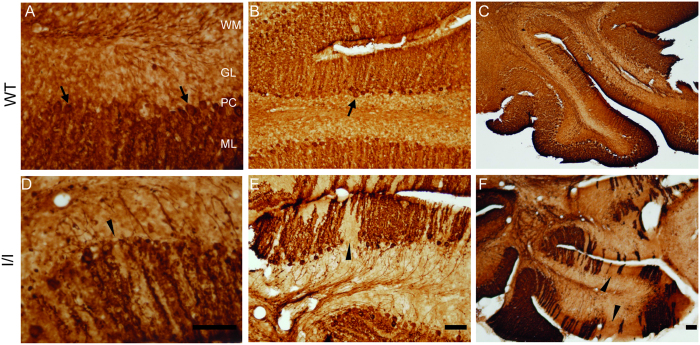
Immunohistochemical analysis of calbindin-D28k in the cerebellum. Coronal cerebellar sections were obtained from (**A**–**C**) 8-week-old wild-type mice and (**D**–**F**) mutant mice. Purkinje cells were immunolabelled (arrows) using an antibody against calbindin-D28K. In mutant mice, these cells showed soma reduction (arrows) (**D**–**F**) compared to WT animals (**A**–**C**), as well as alterations in the dendritic arborisation pattern (arrow heads) (**D**–**F** *vs* **A**–**C**). Abbreviations: ML, molecular layer; PC, Purkinje cells; GL, granular layer; WM, white matter. Scale bar, 100 μm.

Immunohistochemical analyses against calbindin revealed alterations in the cerebellum of mutant mice mainly noticed in the Purkinje cells which displayed a soma reduction together with alterations in the dendritic pattern compared to wild type (Fig. 5).

### Face validity and body weight

The visually detectable symptoms (tremor and unstable gait) of NPC disease appeared in the seventh postnatal week in both I/I and I/P animals. Before that age, it was not possible to distinguish diseased from healthy animals visually. However, we did detect some differences in motor behaviour and cognition at earlier stages (at 5 to 8 weeks). At approximately 7 weeks of age, there was an onset of visible tremors and ataxia in I/I and I/P mice, which showed impaired motor coordination compared to WT animals.

**Figure 6:**
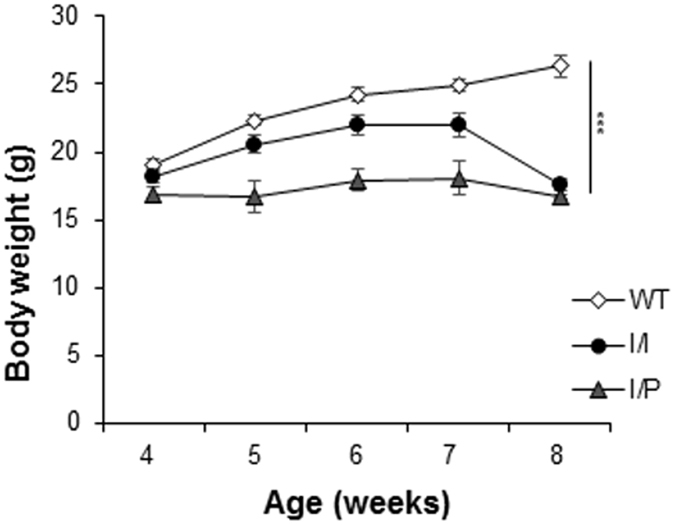
Changes in body weight in I/I and I/P mice compared to WT. ***p < 0.001.

Both I/I and I/P male mice at 4 weeks of age presented similar body weight to WT mice. However, I/I mice showed reduced weight gain until week 7, and a decrease in weight at week 8 when tremor and ataxia were already noticeable. WT animals steadily increased their body weight with time, while I/P mice showed a flat curve for body weight increase [I/I and I/P: genotype effect, F(1,25) = 28.83; p = 0.000 (two-way repeated measures ANOVA)] (Fig. 6).

### Grip strength

Forelimb grip strength (Newtons per gram of body weight) increased with age in the I/I mice [Bonferroni post hoc test for 5 weeks *vs* 8 weeks for forelimbs, p = 0.002; Bonferroni post hoc test for 5 weeks *vs* 8 weeks for all limbs, p = 0.02], being higher in 8-week-old I/I mice than in 8-week-old WT mice [Bonferroni post hoc test for the forelimbs of 8-week-old I/I mice *vs* 8-week-old WT mice, p = 0.003]. In the I/P mice, we detected no differences in grip strength between the genotypes or ages for the forelimbs and all limbs.

### Locomotor activity

**Figure 7:**
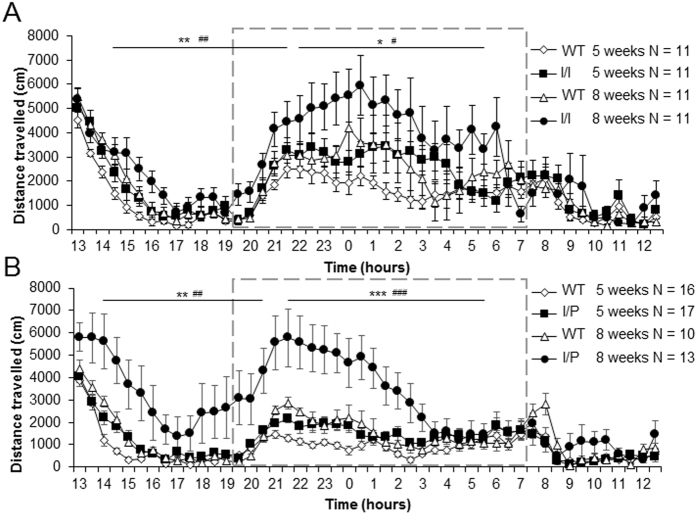
Circadian activity. Distance travelled (in cm) every 30 minutes for 24 hours by (**A**) I/I and (**B**) I/P animals compared to WT. Grey dashed box represents the active phase of the circadian cycle. Data are expressed as mean ± SEM. Genotype effect: *p < 0.05; **p < 0.01. Age effect: ^#^p < 0.05; ^##^p < 0.01.

We analysed locomotor activity at 5 weeks of age, when the motor symptoms were not yet visible, and at 8 weeks, when the mutant animals presented tremor and ataxia. At 5 weeks, both WT and transgenic mice (I/I and I/P) showed novelty-related hyperactivity in the first three to four hours of exposure to a new environment (light phase) [I/I: genotype effect, F(1,42) = 10.42; p = 0.002; I/P: genotype effect, F(1,51) = 8.43; p = 0.005 (two-way repeated measures ANOVA)]. However, I/I and I/P mice travelled a longer distance at 8 weeks than at 5 weeks (Fig. 7A and B, respectively) [I/I: age effect, F(1,42) = 13.83; p = 0.001; I/P: age effect, F(1,51) = 8.37; p = 0.006 (two-way repeated measures ANOVA)], indicating an age-associated increase in activity. Hyperactivity was also detected during the active (dark) phase of the circadian cycle in both transgenic lines, which increased with age [I/I: age effect, F(1,42) = 3.6; p = 0.064; genotype effect, F(1,42) = 4.81; p = 0.034; I/P: age effect, F(1,51) = 17.05; p < 0.001; genotype effect, F(1,51) = 15.01; p < 0.001; genotype × age effect, F(1,51) = 4.50; p = 0.039 (two-way repeated measures ANOVA)].

### Novel object recognition test

**Figure 8:**
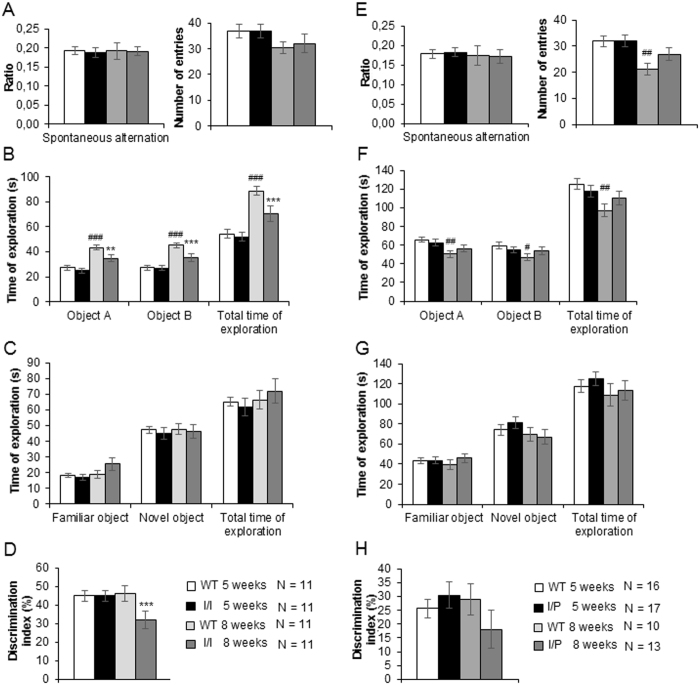
Novel object recognition test in (**A**–**D**) I/I and (**E**–**H**) I/P mice. (**A** and **E**) Habituation session. Spontaneous alternation (expressed as the number of alternations divided by the total number of entries minus 2) and the total number of entries into the arms of the Y maze. Data are expressed as mean ± SEM. (**B** and **F**) Time of exploration during the familiarisation session. (**C** and **G**) Time of exploration during the recognition session. (**D** and **H**) Discrimination index was calculated by subtracting the time spent exploring the familiar object from that spent exploring the novel object and then dividing the sum by the total exploration time, before multiplying it by 100 to obtain a percentage.

The novel object recognition test was used to assess cognition at 5 and 8 weeks of age. In the habituation session, all the mice showed similar percentages of spontaneous alternation, with no significant differences between genotypes or ages. During the habituation session, there were no genotype-dependent differences in exploratory behaviour (number of arm entries) in any of the transgenic mice, but all the mice showed a reduced number of entries at 8 weeks of age that was statistically significant only in the WT animals [Bonferroni post hoc test for the number of entries of 5–week-old WT *vs* 8-week-old WT mice, p = 0.008] (Fig. 8A,E).

In the familiarisation session, 5–week-old I/I mice showed no differences in exploration compared to WT. At 8 weeks, I/I animals showed reduced exploratory behaviour compared to WT mice of the same age [Bonferroni post hoc test for 8-week-old WT *vs* 8-week-old I/I mice for the total time of exploration, p = 0.012] (Fig. 8B). Conversely, the I/P line showed no differences in exploratory behaviour compared to WT (Fig. 8F).

In the test session, there were no differences in exploratory behaviour among all the 5-week-old mice (Fig. 8C,G). However, at 8 weeks of age, both I/I and I/P mice showed impaired memory recognition compared to their WT littermates, which reached statistical significance in the I/I animals [Bonferroni post hoc test for 8-week-old I/I *vs* 8-week-old WT mice for the discrimination index, p = 0.009] (Fig. 8D), but not in the I/P mice (Fig. 8H).

### Elevated plus maze test

**Figure 9:**
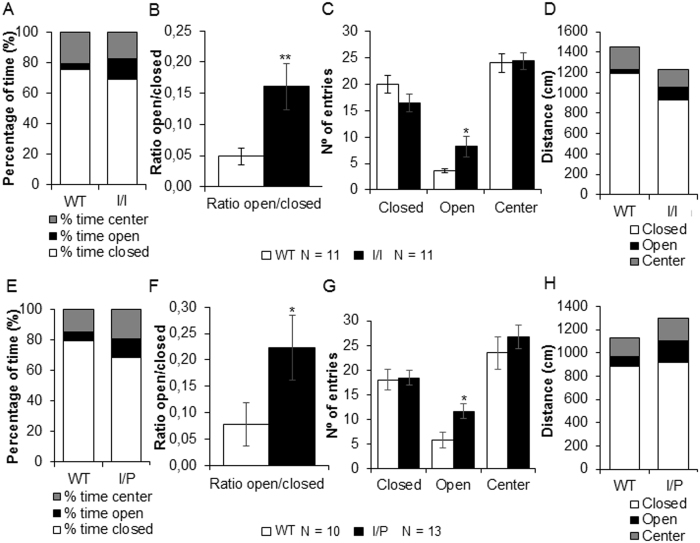
Elevated plus maze test for (**A**–**D**) I/I and (**E**–**H**) I/P mice. (**A** and **E**) Stacked bar chart showing the percentage of time spent in open or closed arms or in the centre of the maze. (**B** and **F**) Ratios of the time spent in the open arms to that spent in the closed arms. (**C** and **G**) Number of entries into the arms. (**D** and **H**) Stacked bar charts showing the distance travelled (cm) in open or closed arms or in the centre of the maze. Data are expressed as mean ± SEM. *p < 0.05; **p < 0.01.

To study anxiety-like behaviour, mice were placed in an elevated plus maze (EPM) for 5 minutes. The percentage of time spent in the open or closed arms of the maze and the ratio between them was calculated as a measure of anxiety. Both I/I and I/P mice spent more time in the open arms compared to WT, suggesting less anxiety-like behaviour [I/I: percentage of time in the open arms (OA) of the maze, genotype, t(1,20) = −2.089; p = 0.050; ratio of time spent in the OA/closed arms (CA) of the maze, genotype, t(1,20) = −2.38; p = 0.010; I/P: percentage of time in the OA, genotype, t(1,22) = −2.56; p = 0.018; percentage of time in the CA, genotype, t(1,22) = 2.29; p = 0.022; ratio of time spent in the OA/CA of the maze, genotype, t(1,22) = −2.25; p = 0.035 (Student’s t-test)] (Fig. 9).

The number of entries, the distance travelled and the speed were recorded for the entire session as a measure of activity. All mutant animals travelled more in the open arms of the maze than in the closed arms [I/I: distance travelled in the OA, genotype, t(1,20) = −2.87; p = 0.013; distance travelled in the CA, genotype, t(1,20) = 2.19; p = 0.040; I/P: distance travelled in the OA, genotype, t(1,22) = −2.37; p = 0.015 (Student’s t-test)]. Both I/I and I/P mice entered the open arms more often than WT animals [I/I: number of entries into the OA, genotype, t(1,20) = −2.31; p = 0.032; I/P: number of entries into the OA, genotype, t(1,22) = −2.53; p = 0.017 (Student’s t-test)], revealing reduced anxiety-like behaviour in the mutant animals.

### Rotarod performance test

**Figure 10:**
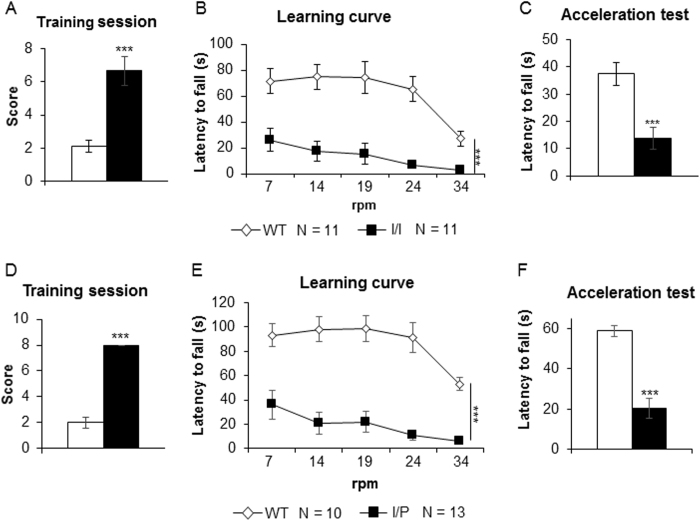
Rotarod motor coordination test for (**A**–**C**) I/I and (**D**–**F**) I/P mice. (**A** and **D**) Training session. Scores indicate the number of times the mice fell off the apparatus at 4 rpm. (**B** and **E**) Latency to fall off at different velocities. (**C** and **F**) Latency to fall off at increasing velocity from 4 to 40 rpm in 1 minute in the acceleration session. Data are expressed as mean ± SEM. ***p < 0.001.

To measure motor coordination, the number of times that the animals needed to stand for 2 minutes on the rod during the rotarod performance test was scored as a percentage (8 sessions was the maximum allowed). Both I/I and I/P mice showed a significantly higher percentage compared to WT [I/I: genotype, t(1,17) = −4.93; p < 0.001; I/P: genotype, t(1,17) = −22.43; p < 0.001 (Student’s t-test)] (Fig. 10). Next, animals were tested at 5 different velocities (7, 14, 19, 24 and 34 rpm), undergoing two trials for each speed. Here, cerebellar learning can be assessed by the repeated testing of an increasingly difficult motor task. Improvement in performance was indicated by a longer latency to fall off the rotarod. All mutant mice were unable to learn, as shown in Fig. 10B and E, whereas WT animals efficiently learnt the task [I/I: genotype effect, F(1,17) = 36.521; p < 0.001; I/P: genotype effect, F(1,17) = 61.04; p = 0.000 (two-way repeated measures ANOVA)]. In the acceleration session (Fig. 10C,F), the latency to fall off was also significantly reduced in mutant mice compared to WT [I/I: genotype, t(1,17) = 6.13; p < 0.001; I/P: genotype, t(1,17) = 6.16; p < 0.001 (Student’s t-test)].

### Hot plate test

**Figure 11:**
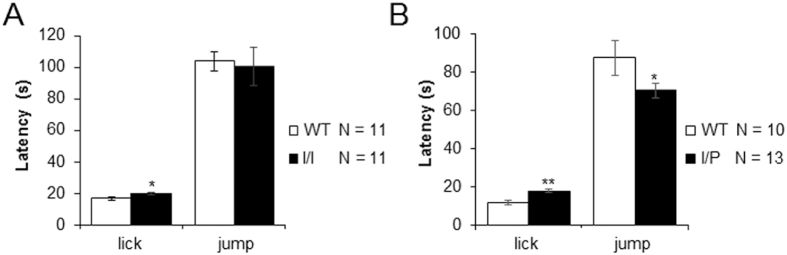
Hot plate test in (**A**) I/I and (**B**) I/P mice compared to WT. Latency to lick front paws and to jump was recorded. Data are expressed as mean ± SEM. *p < 0.05; **p < 0.01.

To assess nociception, mice were placed on a hot plate at 52 ± 0.1 °C and the time of latency to paw licking and jumping was recorded. All mutant animals seemed to have a slightly higher pain threshold than WT (Fig. 11A,B), presenting a longer delay before licking their front paws [I/I: genotype, t(1,20) = −2.18; p = 0.041; I/P: genotype, t(1,20) = −3.01; p = 0.005 (Student’s t–test)]. Moreover, I/P animals also showed a reduced latency to jumping than WT mice [I/P: genotype, t(1,20) = 2.07; p = 0.017] (Fig. 11B).

### Analysis of paw prints

**Figure 12:**
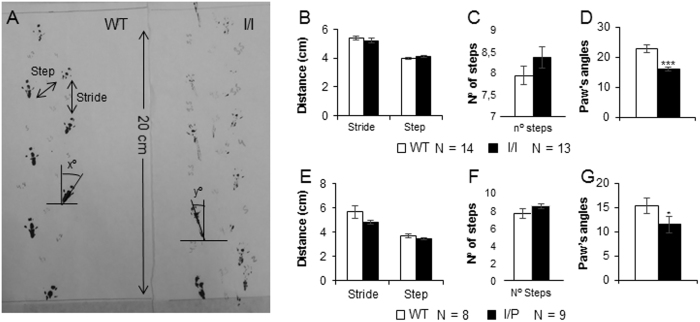
Analysis of paw prints. (**A**) Schematic of the footprints analysed in I/I mice compared to WT. The mean distance between steps and strides (cm) in (**B**) I/I and (**E**) I/P mice. Number of steps recorded in 20 cm of runway in (**C**) I/I and (F) I/P mice. Paw angles measured as degrees in (**D**) I/I and (**G**) I/P mice. Data are expressed as mean ± SEM. *p < 0.05; ***p < 0.001.

The footprint test was used to assess gait and identify any abnormal movements in mice. The number of steps, stride length (distance between consecutive prints made by the same hind paw), step length (distance between consecutive prints made by different hind paws) and the hind paw angle were measured in 8-week-old mice (Fig. 12A). All I/I and I/P mice showed significantly reduced hind paw angles compared to WT animals [I/I: genotype, t(1,25) = 4.82; p < 0.001; I/P: genotype, t(1,20) = 2.08; p = 0.048 (Student’s t-test)]. However, there was no difference between the genotypes regarding the number of steps, the stride or step length (Fig. 12B–G).

## Discussion

Here, we describe the molecular and behavioural characterisation of new NPC mouse models bearing two mutations found in human patients. One of the mutations, c.1920delG (Pioneer (P)) a novel mutation located in exon 12 (out of 25), generates a frameshift with a premature stop codon two residues downstream of the mutation (p.His641ThrfsX2). The other mutation is a deep intronic transition, c.1554-1009 G > A (Imagine (I)), which results in the inclusion of a 194-bp pseudoexon in intron 9 of the *NPC1* gene in the mature mRNA. This incorporation generates a premature stop codon. This mutation was first described by our group in a Spanish patient^13^ and was later found in other Spanish patients^14^. The Addi and Cassi Fund (http://addiandcassi.com/), which helped support the present research, was established in the name of American twins diagnosed with NPC who are compound heterozygotes for these two mutations, c.1920delG and c.1554-1009 G > A. Here, we demonstrate that these mouse models present the main features of the disease. This will enable the development of future *in vivo* therapeutic approaches to eliminate the pseudoexon using viral vectors carrying antisense oligonucleotides, similar to that previously reported at the cellular level^13^.

It was not possible to study the *Npc1*^*pioneer/pioneer*^ model because the birth rate of homozygous mice (2%) was much lower than expected. In other NPC mouse models, such as the *Npc1*^*nih*^ mice, the birth rate of homozygous animals has been reported to be around 16–18%^10^. It is difficult to speculate on the causes of the decreased birth rate of *Npc1*^*pioneer*^ homozygous mice. It is clear that the c.1920delG mutation is severe, since it generates a premature stop codon that produces a truncated protein (if the RNA is not degraded by the nonsense-mediated RNA decay mechanism). However, *Npc1*^*nih*^ mice also carry a premature translation-termination codon (PTC)-producing mutation and have a better birth rate. It may be that the truncated *Pioneer* protein has a specific deleterious effect, which is yet to be elucidated. The mutation is located in the third transmembrane domain, at the beginning of the SSD domain, and it is known that mutations affecting the SSD domain of the NPC1 protein are particularly deleterious, since they correspond to the most severe neurological form of the disease^15^.

The birth rate of the *Npc1*^*imagine*^ homozygous mice was the same as that of the *Npc1*^*nih*^ mice, providing sufficient animals to perform behavioural and molecular tests. This was also true for the *Npc1*^*imagine/pioneer*^ mice. The animals with the pseudoexon-generating *Imagine* mutation are the first NPC mouse models to bear this type of mutation. Thus, they provide a very good opportunity to test treatments based on antisense oligonucleotides.

A very important aspect of this study was to determine whether the mutant mice produced the same aberrant splicing as that observed in human cells^13^. We noted that the main transcript in the brain cortex of *Npc1*^*imagine*^ mice contained the pseudoexon. A second transcript that skipped exon 10 was also observed. Neither of these can produce a functional protein. These two transcripts were also observed in the liver, although two additional faint bands were also present. The size of one of these was consistent with that of the WT transcript, while the other was slightly larger. The NPC1 protein was not detected in the liver of mutant mice, and its levels were reduced in the brain. These total or partial reductions of NPC1 protein levels are consistent with that found in other NPC mouse models^10^,^12^. Thus, the *Imagine* mutation generated a truncated protein that not only altered the protein’s function, but also reduced the total amount of protein, possibly leading to a more severe phenotype.

Lipid accumulation in *Npc1*^*imagine/imagine*^ and *Npc1*^*imagine/pioneer*^ mice was consistent with that found in NPC patients and other NPC mouse models. It is known that NPC patients accumulate different lipids, including cholesterol, sphingomyelin, multiple glycosphingolipids and sphingosine^16^. While cholesterol clearly accumulates in the liver, the main lipids accumulating in the brain are GM2 and GM3 gangliosides^3^. Our models showed an accumulation of cholesterol in the liver and a clear increase in GM2 and GM3 levels in the brain. Liver accumulation of cholesterol has also been observed in other NPC mouse models^16^,^17^, in a range consistent with that obtained in the present study. Moreover, our histopathology results were similar to those observed in other mouse models^18^,^19^,^20^.

The accumulation of GM2 and GM3 in the brain has also been observed in NPC mouse models. For example, Sleat *et al*.^21^ observed an 11- to 15-fold increase in GM2 and GM3 levels in different NPC mouse models, with Maue *et al*.^10^ and Fan *et al*.^22^ reporting similar results. We also observed increased lactosylceramide and decreased monohexosylceramide concentrations, which are in accordance with those reported by Fan *et al*.^22^, who were the first to describe the reduction in monohexosylceramide levels.

Both I/I and I/P mice presented tremor and ataxia at 7–8 weeks of age, mimicking NPC pathological symptoms. Moreover, affected animals showed an initial growth retardation and rapid loss of body weight after 7 weeks. Such a rapid decrease in weight might not be only due to motor disabilities, but also to nutrition or metabolic factors.

Some of our tests were performed at 7–8 weeks of age, when pathological symptoms had become noticeable. Thus, tremor and ataxia may have affected performance in the motor coordination tests. In fact, the rotarod and paw printing tests revealed the expected motor impairment and abnormal gait in both I/I and I/P mice. Motor behaviour is mediated by several brain structures, including the sensorimotor cortex, cerebellum, brain stem and spinal cord. Here, great differences were found in all the parameters tested in the rotarod performance tests that require the normal functioning of the cerebellum, as well as other brain structures. I/I and I/P mice were unable to stand or walk on the rod in both the learning and acceleration sessions, as demonstrated by the markedly lower time of latency to fall compared to WT mice, indicating that motor coordination in mutant mice was dramatically affected at 8 weeks of age. Moreover, the paw printing test showed significant differences in the paw’s angle, suggesting an abnormal gait in both I/I and I/P mice. These results are consistent with those previously described in NPC mouse models^23^.

Impaired motor performance in NPC mouse models has been previously assigned to axonal degeneration and the progressive loss of neurons in the cerebellum. The loss of cerebellar Purkinje cells is often a prominent feature of NPC1 disease in humans^3^,^24^. The loss of these cells is also one of the main features of NPC1 disease in other mouse models, with Purkinje cell loss and degeneration detected by 28–40 days and becoming acute by 60 days of age^25^. These data were consistent with our immunohistochemical results obtained from using an anti-calbindin antibody to identify Purkinje cells. Purkinje cells displayed alterations in I/I mutant mice compared to WT at 8 weeks of age (Fig. 5), which could explain the neurological changes observed in mutant mice.

In our behavioural tests, I/I mice showed higher muscle tone in their forelimbs at an advanced age (8 weeks) than WT animals, suggesting hypertonia in I/I, but not in I/P mice.

The hyperactivity detected in both I/I and I/P mice during the active phase of the circadian cycle (dark phase) was also observed in the EPM test. In the EPM test, mice preferred the safety of the enclosed darker arms, but liked to explore the open arms and poke over the edge. Less anxious mice venture more into the open arms and poke their heads over the edges of the open arms^26^. The total number of entries and total distance travelled in an EPM are considered a useful index of general activity. The total number of entries is also an index of anxiety, with the percentages of entries and time spent in each arm constituting the index of primary anxiety. Therefore, avoidance of the open arms is linked to higher levels of fear^27^. Our results revealed reduced anxiety-like behaviour in both I/I and I/P mice.

In the hot plate test, I/I diseased animals showed a higher latency to lick their front paws, indicating that the NPC mutation in I/I mice could decrease pain sensitivity. On the other hand, I/P mice showed a lower latency to jump compared to WT, suggesting higher sensitivity to pain in their hind paws. In conclusion, motor response to pain was not affected in I/I and I/P mouse models.

In human patients, NPC is characterised by cognitive decline as a function of age. We noted significantly reduced exploratory behaviour in the familiarisation session in 8–week-old I/I mice compared to 8-week-old WT mice. This was not due to increased anxiety since I/I mice showed reduced anxiety-like behaviour in the EPM test. In the test sessions, no differences in exploration were detected between the genotypes in either I/I or I/P mice. However, when the discrimination index was calculated, impaired recognition memory was observed in I/I, but not I/P mice. The impaired learning and memory performance appeared when clinical symptoms were already present at 8 weeks of age. Although the exact processes underlying this ‘recognition memory’ require further elucidation, good performances in the novel object recognition test depend on appropriate functioning of the cortico-hippocampal system^28^. Thus, our results suggest cortico-hippocampal deficits in I/I diseased animals that may not affect I/P mice. This is in line with previous studies in NPC mouse models describing dramatic decreases in the number of neurons and synaptic plasticity in the hippocampus^29^,^30^.

It is difficult to speculate on the differences between the two mouse models, since both mutations produce a premature stop codon in the first half of the open reading frame. In general, the behavioural test results are consistent with those reported in previous studies^23^,^31^. For the time being, there is no specific treatment for NPC. However, we suggest that our mouse model can be used to study possible pharmacological or genetic interventions against NPC using the simple and inexpensive battery of tests applied in the present study or a more extended approach.

In conclusion, despite some differences, we obtained similar results in the lipid accumulation pattern and neurobehavioural tests for both mouse models. Therefore, *Npc1*^*imagine/imagine*^ and *Npc1*^*imagine/pioneer*^ mice are good models for NPC and can be used for further therapeutic trials. Furthermore, these models are the first to carry a pseudoexon-generating mutation and thus, represent an essential tool for treatment trials based on correcting aberrant splicing.

## Methods

### Animals

The heterozygous *Npc1*^*imagine/*+^ and *Npc1*^*pioneer/*+^ mice have a C57BL/6 genetic background, and were generated by the Ozgene company and kindly given to us for this study by the Addi and Cassi Fund (http://addiandcassi.com/). These mice are available at Jackson Laboratory (https://www.jax.org/) for purchase and stud. The heterozygous *Npc1*^*imagine/*+^ mice have one WT allele (containing the wild-type mouse intron 9) and another in which intron 9 of the mouse *Npc1* gene has been replaced by the whole intron 9 of the human *NPC1* gene carrying the mutation c.1554-1009 G > A. In NPC patients, c.1554-1009 G > A produces aberrant splicing that generates a 194-bp pseudoexon. Heterozygous *Npc1*^*imagine/*+^ mice were interbred and generated litters consisting of *Npc1*^*imagine/imagine*^, *Npc1*^*imagine/*+^ and *Npc1*^+*/*+^ mice.

The heterozygous *Npc1*^*pioneer/*+^ mice have a WT allele and another that has a modified exon 12 containing the c.1920delG mutation and a stop codon in the position where translation terminates in the human *NPC1* gene bearing the c.1920delG mutation. Heterozygous *Npc1*^*pioneer/*+^ mice were interbred and generated litters consisting of *Npc1*^*pioneer/pioneer*^, *Npc1*^*pioneer/*+^ and *Npc1*^+*/*+^ mice.

Animals were fed and maintained in the Barcelona Biomedical Research Park (PRBB) animal facility, certified by the Association for Assessment and Accreditation of Laboratory Animal Care (AAALAC-I, reference number 001339). Animals were housed in standard macrolon cages (40 cm long × 25 cm wide × 20 cm high) under a 12-hour light/dark cycle (lights on from 08:00 to 20:00) in controlled environmental conditions of humidity (50–70%) and temperature (22 ± 2 °C), with food and water supplied *ad libitum*. All experimental procedures were approved by the local ethics committee (CEEA-PRBB) and carried out according to the guidelines of local and European regulations.

### Genotype analysis

To genotype the mice, a 1–mm-long tail fragment was cut with a cautery and genomic DNA extracted for use as a template in PCR amplification.

The tail fragment was digested in 300 μl of 50 mM NaOH at 98 °C for 30 minutes. The samples were then vortexed and 30 μl of 1 M Tris-HCl, pH 8, added to neutralise tissue digestion. Afterwards, samples were centrifuged for 6 minutes at 132,000 rpm to separate the non-digested fraction. One μl of the supernatant was used to perform PCR amplification.

Three specific PCR primers (two forward and one reverse) were used to check for the presence of the c.1554-1009 G > A mutation (Imagine). The WT allele was detected by PCR performed with a forward mouse NPC primer and a reverse NPC primer that hybridised with mouse intron 9 and exon 10, respectively (producing a 151-bp fragment). The mutated allele was detected using a forward human NPC primer that hybridised with human intron 9, and the same abovementioned reverse NPC primer (producing a 185-bp fragment). These primers are available upon request. Fragments were separated by 2% agarose gel electrophoresis.

The c.1920delG (Pioneer) mutation was detected by PCR followed by restriction enzyme analysis, since this mutation generates an *Nla*III restriction site. DNA fragments were separated by 2.5% agarose gel electrophoresis. The WT allele produced a 193-bp and a 36-bp fragment, while the mutated allele gave 144-bp, 48-bp and 36-bp fragments.

### RT-PCR transcript analysis

Mice were euthanised with CO_2_ followed by cervical dislocation. The brains and livers were dissected, immediately frozen with liquid nitrogen and stored until use.

Approximately 100 mg of frozen mouse brain and 30 mg of frozen mouse liver were homogenised using TissueRuptor (QIAGEN Science, Maryland, USA) in QIAzol Lysis reagent (QIAGEN) and the RNeasy Lipid Tissue Mini Kit (QIAGEN), following the manufacturer’s recommendations for total RNA isolation. Total RNA was quantified using the NanoDrop 1000 spectrophotometer (NanoDrop Technologies, Wilmington, DE, USA). Reverse transcription was performed with the High-Capacity cDNA Reverse Transcription Kit (Applied Biosystems, Foster City, CA) according to the manufacturer’s instructions.

RT-PCR splicing analysis was performed using a forward primer hybridising with exon 9 and a reverse one hybridising with exon 11. The sizes of the expected fragments, depending on the type of splicing, were: 296 bp if produced by normal splicing; 463 bp if the pseudoexon was included; and 168 bp if exon 10 was skipped. All RT-PCR products were sequenced to confirm their identity.

### Protein lysates and western blot analysis

Tissue lysates were obtained by adding ice-cold RIPA buffer (0.25% deoxycholate w/v, 1% NP40 v/v, 1 M Tris, pH 7.5, 500 mM EDTA, 5 M NaCl and a protease inhibitor cocktail) (500 μl/100 mg tissue) and passing the tissues through a 5-ml syringe equipped with a 21xG needle. Sonication was subsequently applied for two minutes (10-second on/off intervals) to ensure complete homogenisation. Protein concentration was determined using the Lowry method, and 50 μg of lysate were loaded on to 7.5% SDS-PAGE gels, transferred onto an Immobilon-P transfer membrane (Millipore, Billerica, MA, USA) and blocked with 5% non-fat milk in phosphate-buffered saline (PBS) containing 0.2% Tween-20 (PBST) for 1 hour. The membranes were incubated overnight at 4 °C with a primary antibody against NPC1 (ab36983, Abcam, UK) diluted 1:1,000, and then washed. Anti-rabbit IgG antibody (A0545, Sigma-Aldrich, UK) was used as a secondary antibody (diluted 1:10,000). For normalisation, α-tubulin levels were measured with an anti-α-tubulin antibody (T5168, Sigma-Aldrich, UK; diluted 1:8,000). Bands were detected with the LAS-4000 mini system (Fujifilm).

### Liver histology

Mice were sacrificed with CO_2_ and transcardially perfused rapidly with PBS solution, before being fixed with 4% (w/v) paraformaldehyde. Livers were removed and postfixed in the same fixative for 24–48 hours. Fixed samples were paraffin-embedded, sectioned and stained with haematoxylin and eosin (H&E). Samples from 9 WT and 9 *Npc1*^*imagin//imagine*^ mice were analysed.

### Brain histology

Free-floating coronal sections were rinsed in PBS, pH 7.35, and then treated with 0.5% H_2_O_2_ and 10% methanol in PBS for 15 minutes. After that, they were preincubated for 2 hours in a blocking solution (10% foetal bovine serum (FBS), 0.2 mM glycine, 0.1% Triton X-100 in 0.2% PBS-gelatine). Sections were then incubated overnight at 4 °C with the primary polyclonal antibody against calbindin (diluted 1:1000; Swant Inc, Switzerland).

Sections were then sequentially incubated with biotinylated secondary antibody (diluted 1:200; Sigma-Aldrich) for 2 hours at room temperature and then with the avidin-biotin-peroxidase complex (ABC; diluted 1:200; Vector Laboratories, USA). Peroxidase reactions were performed with 0.03% DAB in 0.1 M PB and 0.01% H_2_O_2_, and immunoreacted sections then mounted onto gelatinised slides. The stained sections were examined under a light microscope (Olympus, BX61, Olympus Optical, Hamburg, Germany).

### Cholesterol measurement

Liver tissue was homogenised using TissueRuptor (QIAGEN) in PBS at 5% w/v. Lipids were extracted with a chloroform:methanol (2:1) mixture containing stigmasterol as the internal standard. Samples were derivatised with *N,O*-Bis(trimethylsilyl) trifluoroacetamide (BSTFA) to form the trimethylsilyl derivatives and analysed by gas chromatography-mass spectrometry. Gas chromatography coupled to electron impact (70 eV) mass spectrometry was carried out using a Fisons gas chromatograph (8000 series) coupled to a Fisons MD-800 mass-selective detector. The system was equipped with an HP-5-MS capillary column (30 m × 0.20 mm inner diameter), which was programmed to increase from 120 °C to 315 °C at 5 °C/minute after an initial delay of 2 minutes. Analyses were performed in the selected ion-monitoring mode. The ions selected were those at m/z 129, 458 and 484.

### Sphingolipid determination

Brain tissue was homogenised using TissueRuptor (QIAGEN) in 0.2 M sucrose at 5% w/v. Protein concentration was determined by the Lowry method and a 400-μg protein aliquot used for sphingolipid quantification. Sphingolipid extracts, fortified with internal standards, were prepared as described elsewhere^32^ and different groups of lipids, including ceramides, sphingomyelins and glycosphingolipids, were analysed by liquid chromatography-mass spectrometry (LC/MS), as reported previously^33^ with minor modifications. The LC/MS analysis consisted of a Waters Acquity UPLC system connected to a Waters LCT Premier orthogonal accelerated time-of-flight mass spectrometer (Waters), operated in positive and negative electrospray ionisation modes. Full scan spectra from 50 to 1500 Da were acquired, and individual spectra were summed to produce data points every 0.2 seconds. Mass accuracy and reproducibility were maintained using an independent reference spray via the LockSpray interference. The analytical column was a 1.7-μm ethylene-bridged hybrid (C8 Acquity UPLC column, Waters), measuring 100 × 2.1 mm (inner diameter). The two mobile phases were MeOH:HCOOH (998:2, v/v) for phase A and water:HCOOH (998:2, v/v) for phase B, both containing 2 mM ammonium formate. The column was maintained at 30 °C. Quantification was carried out using the extracted ion chromatogram of each compound, using 50-mDa windows. The linear dynamic range was determined by injecting standard mixtures. The positive identification of compounds was based on accurate mass measurement with an error of <5 ppm and its LC retention time, compared with that of a standard ( ± 2%).

### Behavioural characterization

Male WT and the *Npc1*^*imagine/imagine*^ and *Npc1*^*imagine/pioneer*^ mice were studied from 21 to 60 days of age. Each experiment used between 6 and 14 animals from each group.

### Grip strength tests

On the test day, each mouse was weighed and singly housed in a holding cage for the duration of the experiment. After all the animals had been weighed, each mouse was tested for grip strength. A metal wire with a diameter of 3 mm was used as the grip bar. The mouse was held near the base of its tail and lowered towards the bar until it gripped the bar with both forepaws first and all limbs afterwards. The mouse was then gently pulled away from the bar at a steady rate of about 2.5 cm/second until the bar was released. Peak force was automatically recorded in Newtons by the apparatus. Data were recorded, and four additional trials were immediately performed. Finally, the average maximal grip force was calculated as Newtons per gram of body weight.

### Hot plate tests

To assess nociception, mice were placed on a hot plate at 52 ± 0.1 °C surrounded by a plastic cylinder (19 cm in diameter and 19 cm high), with latency to paw licking and jumping manually recorded. A maximum latency of 180 seconds was established to avoid tissue damage.

### Circadian activity

Circadian activity was measured using activity cages (TSE, Germany) equipped with infrared photobeam detectors for analysis of horizontal activity. The test was performed under low non-aversive light conditions (50 lux) to avoid stressful stimuli. The total distance travelled every 30 minutes was recorded for 24 hours. Mice were individually housed in standard macrolon cages (40 cm long × 25 cm wide × 20 cm high) for 24 hours.

### Rotarod performance test

The rotarod apparatus was used to measure fore- and hindlimb motor coordination and balance. The protocol consisted of a training session at a constant speed (4 rpm) for a maximum of 60 seconds. Next, animals were tested at 5 different velocities (7, 14, 19, 24 and 34 rpm) for a maximum of 120 seconds, undergoing two trials at each speed with a 5-minute interval between each trial. The time of latency to fall off the apparatus was recorded. One hour later, the mice underwent two trials under accelerating conditions at speed levels increasing from 4 to 40 rpm over a 1-minute period. The mean latency to fall off the rotarod was recorded and used in subsequent analysis.

### Analysis of paw prints

The footprint test was used to assess gait and identify any abnormal movements in mice. To obtain footprints, the hind paws of the mice were coated with non-toxic black paint. The animals were allowed to walk along a runway 50 cm long and 10 cm wide (with 10-cm-high walls) in an enclosed box. All the mice performed the test twice and the average was calculated. A clean sheet of white paper was placed on the floor of the runway for each run. The paw print patterns were analysed for the following (all measured in cm): (1) stride length, measured as the average length of forward strides; (2) step length, measured as the average distance between the left and right hind footprints that was determined by measuring the perpendicular distance of a given step to a line connecting its opposite preceding and proceeding steps; and (3) the angle of each hind footprint. For each step parameter, three values were measured from each run, excluding paw prints made at the beginning and end of the run where the animal was initiating and finishing movement, respectively. The mean value for each set of three values was used in subsequent analysis.

### Elevated plus maze tests

To study anxiety-like behaviour, mice were placed in an elevated plus maze consisting of a black Plexiglas apparatus with four arms (29 cm long × 5 cm wide) forming a plus shape and a central square (5 cm × 5 cm). Two opposite arms were delimited by vertical walls (closed arms) and the other two arms had unprotected edges (open arms). The maze was elevated 40 cm above the floor and placed under indirect light (100 lux). At the beginning of a 5-minute session, each mouse was placed in the central area, facing one of the open arms. A video-tracking camera recorded the experiment and we analysed the time spent, number of entries, speed and distance travelled in the different areas.

### Novel object recognition tests

This test evaluates recognition memory using previously explored objects, an activity that depends on normal hippocampal functioning. Novel object recognition was examined in a Y maze apparatus, which consisted of three arms (each arm measuring 30 cm × 5 cm × 6 cm) forming a Y shape, thus restricting the area where mice were allowed to move and increasing the amount of time for exploration. The protocol consisted of three sessions.

In the habituation session, animals were habituated for 8 minutes to the Y maze. The number of entries into the three arms and the percentage of spontaneous alternation were measured. Spontaneous alternation was considered to have occurred when mice entered previously unexplored arms consecutively. The percentage of spontaneous alternations was calculated as the number of alternations divided by the total number of entries minus 2.

In the familiarisation session, mice were placed in the Y maze again after 24 hours, this time the Y maze contained two identical plastic objects located at the ends of two of the arms. All mice were placed at the end of the arm without the plastic object. The time spent exploring the two objects was recorded for 8 minutes.

In the test session, mice were presented with two objects for 8 minutes after 24 hours, one of which was the same as that used in the familiarisation session while the other was a novel object. A discrimination index was calculated by subtracting the time spent exploring the familiar object from that spent exploring the novel object and then dividing the sum by the total exploration time, before multiplying it by 100 to obtain a percentage. The position of the novel object was varied between the animals.

The arena and objects were thoroughly cleaned between each animal to avoid olfactory cues. All exploration measures were recorded manually by an experimenter blinded to genotype and treatment. Exploratory behaviour was defined as the animal directing its nose towards the object at a distance of less than 2 cm. Sitting on or resting against the object was not considered exploration.

## Additional Information

**How to cite this article:** Gómez-Grau, M. *et al*. New murine Niemann-Pick type C models bearing a pseudoexon-generating mutation recapitulate the main neurobehavioural and molecular features of the disease. *Sci. Rep.* **7**, 41931; doi: 10.1038/srep41931 (2017).

**Publisher's note:** Springer Nature remains neutral with regard to jurisdictional claims in published maps and institutional affiliations.
